# Supplementary material for: Stat-tracks and mediotypes: powerful tools for modern ichnology based on 3D models
Source: PeerJ. 2018 Jan 11;6:e4247. doi: 10.7717/peerj.4247 (PMC5767334; doi:10.7717/peerj.4247)
Supplement: Supplemental Information 1 — The supplemental file includes information about the methodologies used in this paper to collect and analyse three-dimensional data and more information on the geological context of the studied areas, i.e., the Laetoli tracksite (Tanzania) and the Ajoie ichnocoenosis (Switzerland). [file peerj-06-4247-s001.docx]

**Supplemental Information**

**1 Material and Methods**

**1.1 Institutional abbreviations**

All tracks from the Late Jurassic of the Canton Jura were excavated by the Palaeontology A16 (PALA16). They are uniformly labelled with an acronym of the tracksite, the track level, the trackway type and identifier, and the track number. SCR: Courtedoux—Sur Combe Ronde tracksite; TCH: Courtedoux—Tchâfouè tracksite; BSY: Courtedoux—Bois de Sylleux tracksite. Therefore, e.g., SCR1500-T1-L8 refers to the left pes 8 (L8) of the theropod trackway 1 (T1) from level 1500 of the Courtedoux—Sur Combe Ronde tracksite (SCR); BSY1040-S12-LM1 refers to the left manus 1 (LM1) of the sauropod trackway 12 of level 1040 of the Courtedoux—Bois de Sylleux tracksite. These tracks are part of the PALA16 collection, which will be integrated in 2019 in the collection of the JURASSICA Muséum.

Hominin tracks from the Laetoli tracksite do not have a unique labelling system, and the tracks reported here are named after the publication where they are described. For more information please refer to the cited publications.

**1.2 Three-dimensional data**

Digital 3D track data can be obtained from a range of optical laser scanners and increasingly via digital photogrammetry (Falkingham, 2012; Bennett & Morse, 2014).

Bennett et al. (2013) provide a comparative review of data derived from optical laser scanners and photogrammetry concluding that while the former gives more accurately scaled results the latter is operationally much easier.

The data used in this paper have been derived from a variety of sources. In 2011, some of the specimens used in this study were laser-scanned at a sub-millimetric resolution (generally 0.1 mm) with a FARO Platinum Scanarm hand-scanner operated in the field, before removal and/or destruction of specimens in question. The best specimens were collected and are now part of the PalA16 collection, and in 2016 were digitized through photogrammetric processes. The photogrammetric 3D models were obtained using a Canon EOS 70D, 20 Mpixel camera, equipped with a Canon 10-18mm STS or a Canon 18-135mm STS lenses. Models were created using Agisoft Photoscan Pro (v. 1.2.4 and v. 1.2.5; www.agisfot.com) following the procedures of Mallison & Wings (2014). The accuracy of the models ranges between 0.1 and 0.03 mm, and resolution is always sub-millimetric. Scaling and alignment was made in Photoscan Pro. The models were then downscaled to a 2 million faces mesh to ease computation. The human tracks presented here were scanned either in field or from first generation museum casts using a Konica-Minolta Vi-900 optical laser scanner.

Track registration in this paper is done via the freeware DigTrace (www.digtrace.co.uk) which caters for the registration of tracks, comparison and computation of measures of central tendency in a different manner (see also: Bennett et al., 2016a,b).

DigTrace is compiled in Python and facilitates a complete track processing workflow, from loading raw input files in a variety of formats, performing basic measurements, via registration of a collection of prints through landmark matching, to producing a set of statistics describing the registered tracks (Figure 1). It also has a facility for creating 3D models via photogrammetry. The registration process in DigTrace requires the user to denote one of the tracks as the ‘master’ with which all the remaining tracks are aligned, and to define a set of corresponding landmarks (effectively matching points) for each of these tracks. Selection of the master is normally guided by identifying the track that is most topologically complete. Landmarks can be placed on the basis of either formally anatomically-defined points, or more commonly informally based on points of recurrence (i.e. matching the same topological point on the two tracks). These landmarks can also be complemented by ‘geometrical’ landmarks, located, for example, between defined landmarks. Once an optimum registration is achieved the software computes a transformation of the registered track to align it with the master, by minimising the mean squared deviation between the landmark coordinates in the xy-plane. This transformation is implemented for numerical stability using the Kabsch (1976). Once a series of tracks have been registered the software then samples the stacked or registered tracks at various resolutions (0.25, 0.5 and 1.0 mm) to compute a frequency distribution of values for each point from which measures of central tendency can be computed and displayed visually (i.e. display a mean or median track).


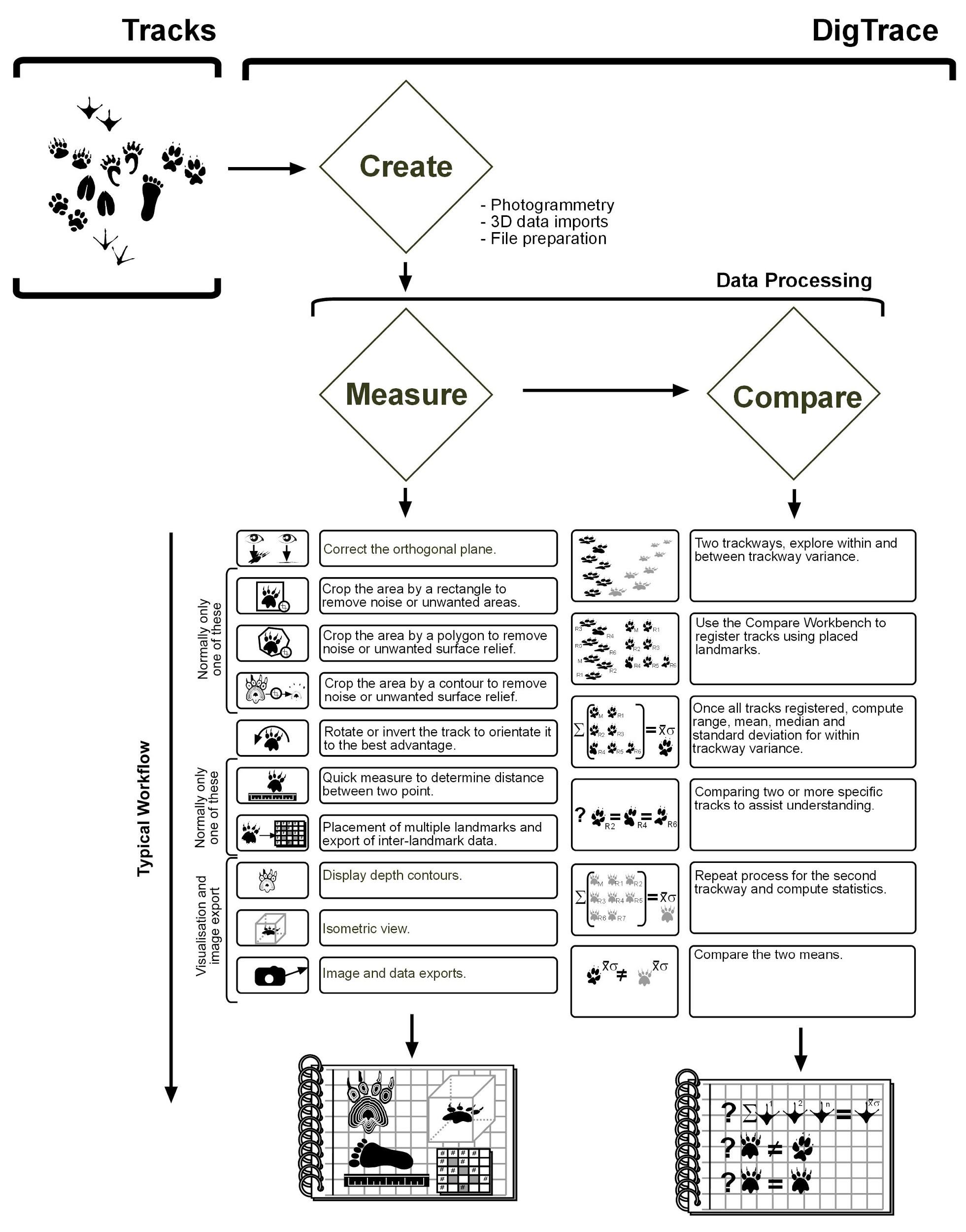


**Figure 1.** Workflow and tools available in DigTrace (www.digtrace.co.uk). The freeware is formulated around three workbenches, create, measure and compare, and allows the complete analysis of 3D tracks from data capture via photogrammetry through to the computation of mean tracks from a trackway or population.

**2 Geological and sedimentological context**

**2.1 Ajoie district dinosaur tracksites**

Since the first discoveries in 2002, the tracksites along Federal Highway A16 have been thoroughly excavated and documented. More than 17,000 m^2^ were unearthed and 13,905 tracks, including 235 sauropod and 405 tridactyl trackways were unearthed and documented. About 520 m^2^ of tracks and trackways were casted and around 900 original tracks (ca. 230 m^2^) were recovered and are stored in the collection of the PALA16.

The studied tracks and trackways come from different track-bearing laminite intervals (of the Reuchenette Formation (Marty, 2008; Comment, Ayer & Becker, 2011). The Reuchenette formation can be precisely dated with ammonites to the Boreal *mutabilis*, respectively Tethyan *acanthicum*, ammonite zones, i.e. early Late Kimmeridgian or about 152.7 to 152.01 Ma (Marty et al., 2003; Jank, Meyer & Wetzel, 2006; Jank, Wetzel & Meyer, 2006; Comment, Ayer & Becker, 2011; Comment et al., 2015). Some of these ammonites were found in layers very close to the dinosaur track-bearing levels. The age assignment is also confirmed with ostracods (Schudack et al., 2013). The Late Kimmeridgian Jura carbonate platform was at a palaeolatitude of around 30° N, at the threshold between the Paris Basin to the northwest and the Tethys Ocean to the south and thus influenced by both the Tethyan and Boreal realms (e.g., Ziegler, 1988; Thierry, 2000; Thierry et al., 2000; Jank, Wetzel & Meyer, 2006). The recurrent presence of dinosaur tracks and emersive phases during the Late Jurassic testifies – at least during sea-level lowstands – prolonged periods of emersion of the Jura carbonate platform and connections with the larger terrestrial landmasses of the London-Brabant Massif in the northeast and/or the Massif Central in the southwest (Jank, Meyer & Wetzel, 2006; Marty, 2008; Marty & Meyer, 2013).

The track-bearing laminite intervals are tabular and platy, thinly-bedded marly limestones, which locally have a slightly stromatolitic appearance and have intercalations of thin layers of calcareous marls (Marty, 2008; Marty et al., 2010). Generally, the microfacies of the laminites is quite homogeneous and can be described as mudstone to wackestone *sensu* Dunham (1962), or dolobiopelmicrite *sensu* Folk, (1962). The most common biogenic sedimentary structures are (microbial) lamination and invertebrate burrows (Marty, 2008).

The track-bearing laminites were deposited in inter- to supratidal flat or supratidal marsh palaeoenvironments, characterized by an exposure index higher than 60–90% (Marty, 2008). This is indicated by macroscopic (stromatolitic lamination, desiccation cracks, wave ripples, invertebrate burrows) and microscopic (e.g., cryptmicrobial lamination, fenestrae, brecciation) sedimentological features (Marty, 2008; Marty & Pacton, 2009). Marty (2008) suggested that this supratidal-flat palaeoenvironment was located several hundred meters away from the coastline towards the open marine realm or an internal lagoon, that for most of the time was characterized by restricted and hostile conditions, which may have been interrupted by occasional wetting due to periods of rain or storm surges, and that during or rather at the end of such periods of wetting, tracks were recorded.

The intermediate track levels with a thickness of around 1 m and at least 15 track-bearing levels are the track-richest interval, whereas the upper track levels are about 30-40 cm thick and contain only 2–3 track levels (Marty et al., 2007). The intermediate levels are suggested to represent 1–2 elementary sequences of each 20 kyr. The sequence boundary Kim4 was placed in the intermediate levels by Colombié & Rameil (2007), but probably corresponds to the upper dinosaur track levels, which in turn likely represent one elementary sequence.

**2.2 Laetoli tracksite**

The Laetoli tracksites are located on the margin of the Eyasi Plateau at the southern margin of the Serengeti Plains, about 20 km north of Lake Eyasi, Tanzania. Despite the huge extension (more than 1000 km^2^) of the fossiliferous outcrops of the Laetoli and Ndolanya Beds, most of the fossils were collected in a relatively restricted area (circa 100 km^2^) along the Garusi, Gadjingero, Nompopong and Olaitole River valleys, representing the Laetoli site (Hay, 1987; Harrison & Kweka, 2011). The Laetoli Beds are probably derived from tephra erupted from the now extinct volcano of Satiman, about 20 km east of Laetoli (Hay, 1987; Ditchfield & Harrison, 2011). The Beds, which represent the base of the sedimentary sequence, are more than 123 m thick and are divisible into two main lithological units (Hay, 1987; Ditchfield & Harrison, 2011): the Lower unit (around 64 m thick) consists mainly of aeolian tuffs interbedded with primary fall-out tuffs and water-worked tuffaceous sediments, and the Upper unit (44–59 m thick) consists of a series of aeolian and fall-out tuffs (Hay, 1987; Ditchfield & Harrison, 2011), and it is topped by the Yellow Marker Tuff. The Upper unit, is then subdivided by other marker tuffs (Tuffs 1-8), which allows a good correlation and precise dating (Deino (2011) dated Tuff 8 to 3.36 Ma).

The more notorious hominin footprints, the G tracksite, from Locality 8 (Harrison & Kweka, 2011) were left on the Footprint Tuff (Tuff 7) level, which also carries most of the Laetoli ichnites (Harrison & Kweka, 2011; Masao et al., 2016). Despite some stratigraphical correlation is possible, the recent findings (Sites L8, M9, M10 and TP2) described by (Masao et al., 2016), cannot be unambiguously correlated with the main Laetoli sites and it is not possible to establish if the sites belong to the same stratigraphic level.

**References**

Belvedere M. 2008. Ichnological researches on the Upper Jurassic dinosaur tracks in the Iouaridène area (Demnat, Central High-Atlas, Morocco).

Bennett MR., Falkingham P., Morse SA., Bates K., Crompton RH. 2013. Preserving the Impossible: Conservation of Soft-Sediment Hominin Footprint Sites and Strategies for Three-Dimensional Digital Data Capture. *PLoS ONE* 8. DOI: 10.1371/journal.pone.0060755.

Bennett MR., Harris JWK., Richmond BG., Braun DR., Mbua E., Kiura P., Olago D., Kibunjia M., Omuombo C., Behrensmeyer AK., Huddart D., Gonzalez S. 2009. Early hominin foot morphology based on 1.5-million-year-old footprints from Ileret, Kenya. *Science* 323:1197–1201. DOI: 10.1126/science.1168132.

Bennett MR., Morse SA. 2014. *Human Footprints: Fossilised Locomotion?* Cham: Springer International Publishing. DOI: 10.1007/978-3-319-08572-2.

Bennett MR., Reynolds SC., Morse SA., Budka M. 2016a. Laetoli’s lost tracks: 3D generated mean shape and missing footprints. *Scientific Reports* 6:21916. DOI: 10.1038/srep21916.

Bennett MR., Reynolds SC., Morse SA., Budka M. 2016b. Footprints and human evolution: Homeostasis in foot function? *Palaeogeography, Palaeoclimatology, Palaeoecology* 461:214–223. DOI: 10.1016/j.palaeo.2016.08.026.

Berge C., Penin X., Pellé É. 2006. New interpretation of Laetoli footprints using an experimental approach and Procrustes analysis: Preliminary results. *Comptes Rendus - Palevol* 5:561–569. DOI: 10.1016/j.crpv.2005.09.001.

Castanera D., Pascual C., Razzolini NL., Vila B., Barco JL., Canudo JI. 2013. Discriminating between medium-sized tridactyl trackmakers: tracking ornithopod tracks in the base of the cretaceous (Berriasian, Spain). *PLoS ONE* 8:e81830. DOI: 10.1371/journal.pone.0081830.

Colombié C., Rameil N. 2007. Tethyan-to-boreal correlation in the Kimmeridgian using high-resolution sequence stratigraphy (Vocontian Basin, Swiss Jura, Boulonnais, Dorset). *International Journal of Earth Sciences* 96:567–591. DOI: 10.1007/s00531-006-0117-3.

Comment G., Ayer J., Becker D. 2011. Deux nouveaux membres lithostratigraphiques de la Formation de Reuchenette (Kimméridgien, Ajoie, Jura suisse) – Nouvelles données géologiques et paléontologiques acquises dans le cadre de la construction de l’autoroute A16 (Transjurane). *Swiss Bulletin for Applied Geology* 16:3–24. DOI: 10.5169/seals-327738.

Comment G., Lefort A., Koppka J., Hantzpergue P. 2015. Le Kimméridgien d’Ajoie (Jura, Suisse): lithostratigraphie et biostratigraphie de la Formation de Reuchenette. *Revue de Paléobiologie* 34:161–194. DOI: 10.5281/zenodo.34341.

Deino AL. 2011. ^40^Ar/^39^Ar Dating of Laetoli, Tanzania. In: Harrison T ed. *Paleontology and Geology of Laetoli: Human Evolution in Context Vol.1*. Springer Netherlands, 77–97. DOI: 10.1007/978-90-481-9956-3_4.

Ditchfield P., Harrison T. 2011. Sedimentology, Lithostratigraphy and Depositional History of the Laetoli Area. In: *Springer Netherlands*. 47–76. DOI: 10.1007/978-90-481-9956-3_3.

Dunham RJ. 1962. Classification of carbonate rocks according to depositional texture. In: Ham, W.E. (Ed.) . In: Ham WE ed. *Classification of Carbonate Rocks*. American Association of Petroleum Geologists, Memoir 1, 108–121.

Falkingham PL. 2012. Acquisition of high resolution three-dimensional models using free, open-source, photogrammetric software. *Palaeontologia Electronica* 15:1T:15p.

Folk RL. 1962. Spectral subdivision of limestone types. In: Ham WE ed. *Classification of Carbonate Rocks*. American Association of Petroleum Geologists, Memoir 1, 62–64.

Hammer Ø., Harper DAT. 2007. *Paleontological Data Analysis*. Malden, MA, USA: Blackwell Publishing. DOI: 10.1002/9780470750711.

Harrison T., Kweka A. 2011. Paleontological Localities on the Eyasi Plateau, Including Laetoli. In: *Springer Netherlands*. 17–45. DOI: 10.1007/978-90-481-9956-3_2.

Hatala KG., Roach NT., Ostrofsky KR., Wunderlich RE., Dingwall HL., Villmoare BA., Green DJ., Harris JWK., Braun DR., Richmond BG. 2016. Footprints reveal direct evidence of group behavior and locomotion in *Homo erectus*. *Scientific reports* 6:28766. DOI: 10.1038/srep28766.

Hay RL. 1987. Geology of the Laetoli area. In: Leakey M, Harris J eds. *Laetoli: A Pliocene Site in Northern Tanzania*. Oxford: Clarendon, 23–47.

Jank M., Meyer CA., Wetzel A. 2006. Late Oxfordian to Late Kimmeridgian carbonate deposits of NW Switzerland (Swiss Jura): stratigraphical and palaeogeographical implications in the transition area between the Paris Basin and the Tethys. *Sedimentary Geology* 186:237–263. DOI: 10.1016/j.sedgeo.2005.08.008.

Jank M., Wetzel A., Meyer CA. 2006. A calibrated composite section for the Late Jurassic Reuchenette Formation in northwestern Switzerland (?Oxfordian, Kimmeridgian *sensu gallico*, Ajoie-Region). *Eclogae Geologicae Helvetiae* 99:175–191. DOI: 10.1007/s00015-006-1187-8.

Kabsch W. 1976. A solution for the best rotation to relate two sets of vectors. *Acta Crystallographica Section A* 32:922–923. DOI: 10.1107/S0567739476001873.

Lallensack JN., van Heteren AH., Wings O. 2016. Geometric morphometric analysis of intratrackway variability: A case study on theropod and ornithopod dinosaur trackways from Münchehagen (Lower Cretaceous, Germany). *PeerJ* 4:e2059. DOI: 10.7717/peerj.2059.

Mallison H., Wings O. 2014. Photogrammetry in paleontology - A practical guide. *Journal of Paleontological Techniques* 12:1–31.

Marty D. 2008. Sedimentology, taphonomy, and ichnology of Late Jurassic dinosaur tracks from the Jura carbonate platform (Chevenez—Combe Ronde tracksite, NW Switzerland): insights into the tidal-flat palaeoenvironment and dinosaur diversity, locomotion, and palaeoecolog. *GeoFocus* 21:1–278.

Marty D., Ayer J., Becker D., Berger JP., Billon-Bruyat JP., Braillard L., Hug WA., Meyer CA. 2007. Late jurassic dinosaur tracksites of the Transjurane highway (Canton Jura, NW Switzerland): overview and measures for their protection and valorisation. *Bulletin fuer Angewandte Geologie* 12:75–89.

Marty D., Belvedere M., Meyer CA., Mietto P., Paratte G., Lovis C., Thüring B. 2010. Comparative analysis of Late Jurassic sauropod trackways from the Jura Mountains (NW Switzerland) and the central High Atlas Mountains (Morocco): implications for sauropod ichnotaxonomy. *Historical Biology* 22:109–133. DOI: 10.1080/08912960903503345.

Marty D., Hug W., Iberg A., Cavin L., Meyer C., Lockley M. 2003. Preliminary Report on the Courtedoux Dinosaur Tracksite from the Kimmeridgian of Switzerland. *Ichnos* 10:209–219. DOI: 10.1080/10420940390256212.

Marty D., Meyer CA. 2013. A bridge over troubled water – The continuous record of terrestrial vertebrates from the Oxfordian to the Berriasian in the Jura Mountains. In: *The Palaeontology Newsletter*. Zurich, 48.

Marty D., Pacton M. 2009. Formation and preservation of Late Jurassic dinosaur track-bearing tidal-flat laminites (Canton Jura, NW Switzerland) through microbial mats. In: Billon-Bruyat J-P, Marty D, Costeur L, Meyer CA, Thüring B eds. *5th International Symposium on Lithographic Limestone and Plattenkalk — Abstracts and Field Guides.International Symposium on Lithographic Limestone and Plattenkalk — Abstracts and Field Guides.* Porrentruy: Société jurassienne d’émulation, actes 2009 bis, 56–58.

Masao FT., Ichumbaki EB., Cherin M., Barili A., Boschian G., Iurino DA., Menconero S., Moggi-Cecchi J., Manzi G. 2016. New footprints from Laetoli (Tanzania) provide evidence for marked body size variation in early hominins. *eLife* 5:e19568. DOI: 10.7554/eLife.19568.

Rodrigues LA., Santos VF dos. 2004. Sauropod Tracks – a geometric morphometric study. In: Elewa AMT ed. *Morphometrics*. Berlin, Heidelberg: Springer-Verlag, 129–142.

Schudack U., Schudack M., Marty D., Comment G. 2013. Kimmeridgian (Late Jurassic) ostracods from Highway A16 (NW Switzerland): taxonomy, stratigraphy, palaeoecology, and palaeobiogeography. *Swiss Journal of Geosciences* 106:371–395.

Thierry J. 2000. Early Kimmeridgian. In: Dercourt J, Gaetani M, Vrielvynck B, Barrier E, Biju-Duval B, Brunet MF, Cadet JP, Crasquin S, Sandulescu M eds. *Atlas Peri-Tethys, Palaeogeographical maps — Explanatory Notes*. Paris, 85–97.

Thierry J., Barrier E., Abbate E., Alekseev AS., Ait-Salem H., Bouaziz S., Canerot J., Georgiev G., Guiraud R., Hirsch F., Ivanik M., Le Metour J., Le Nindre YM., Medina F., Mouty M., Nazarevich B., Nikishin AM., Page K., Panov DL., Pique A., Poisson A., Pique A., Poisson A., Sandulescu M., Sapunov IG., Seghedi A., Soussi M., Tchoumatchenko P V., Vaslet D., Vishnevskaya V., Volozh YA., Voznenezenski A., Walley CD., Wong TE., Ziegler M., Barrier E., Bergerat F., Bracene R., Brunet MF., Cadet JP., Guezou JC., Jabaloy A., Lepvrier C., Rimmele G., De Wever P. 2000. Map 10: Early Kimmeridgian (146-144 Ma). In: Dercourt J, Gaetani M, Vrielvynck B, Barrier E, Biju-Duval B, Brunet M, Cadet J, Crasquin S, Sandulescu M eds. *Atlas Peri-Tethys Palaeogeographical maps*. Paris: CCGM/CGMN (Commission for the Geological Map of the World),.

Ziegler PA. 1988. Evolution of the Arctic North Atlantic and the Western Tethys. *Memoir of the American Association of Petroleum Geologist* 43:1–198.
