# Supplementary material for: Stat-tracks and mediotypes: powerful tools for modern ichnology based on 3D models
Source: PeerJ. 2018 Jan 11;6:e4247. doi: 10.7717/peerj.4247 (PMC5767334; doi:10.7717/peerj.4247)
Supplement: Supplemental Information 2 — The document includes the link to all the repositories where the raw data used in this work are located. [file peerj-06-4247-s002.pdf]

Raw data and software repositories

- DigTrace is freeware and can be downloaded at www.digtrace.co.uk

- Most of the tracks used for the comparison already published.

    - *Jurabrontes curtedulensis* (in: http://dx.doi.org/10.1080/08912963.2017.1324438):
    - http://dx.doi.org/10.6084/m9.figshare.4029291 and http://dx.doi.org/10.6084/m9.figshare.4029285

    - *Megalosauripus transjuranicus* (in: https://doi.org/10.1371/journal.pone.0180289): https://doi.org/10.6084/m9.figshare.4036584.v2

    - *Megalosauripus teutonicus*: https://doi.org/10.6084/m9.figshare.4029306 (in http://dx.doi.org/10.1080/08912963.2017.1324438)

    - Laetoli tracks: http://footprints.bournemouth.ac.uk/archive/Laetoli/

    - Tracks from Masao et al 2016 http://morphosource.org/Search/Index?search=laetoli

- Unpublished material is accessible here: https://figshare.com/s/f842e98af82bc911ed4c (*M. transjuranicus* mean mediotype, small and tiny sauropod tracks and mediotypes)
